# Positioning Food Cultures: ‘Alternative’ Food as Distinctive Consumer Practice

**DOI:** 10.1177/0038038515585474

**Published:** 2015-06-15

**Authors:** Jessica Paddock

**Affiliations:** Sustainable Consumption Institute, University of Manchester, UK

**Keywords:** alternative food, class, consumption, culture, distinction

## Abstract

Many sociological studies to date have explored the role of food in marking distinctions between groups. Less well understood is how ‘alternative’ means of food consumption become figured in such relations. Drawing on accounts of food practice derived from 20 in-depth interviews and a two-year period of participant observation, this article considers the role of class culture in the practice of alternative food consumption. As participants speak their position, expressions of class arise through discussions of food practice. Having explored how food plays a part in marking boundaries of distinction between foods ‘for us’ and ‘for them’, we are reminded that in reproducing certain ideas about proper eating, we confine our imagining of alternative food futures to a limited politics of the possible. The article highlights implications for future development of equitable alternatives to conventional foodways.

## Introduction

As awareness of the environmental effects of advanced capitalism grows, we witness the progression of ‘ethical’ (Harrison et al., 2005), ‘alternative’ (Goodman and Goodman, 2009) or ‘sustainable’ (Jackson, 2006) consumption movements. Here, the promise of fairer conditions of trade between consumers and producers has also come to ground food in questions that seek to realise global social justice (Lyon and Moberg, 2010), while shortening food supply chains and grounding them within local economies has become a core focus for the wider project of sustainable development (Arce and Marsden, 1993). Although each movement appeals to consumers’ ethical dispositions, their sense of what is moral, good and responsible, they do so with differing emphasis upon abstinence from or modification of existing consumption practices (Sassatelli and Davolio, 2010). For Littler (2009), ethical consumption is considered an act of sanctimonious shopping, which she describes as a means of extolling one’s moral virtues and displaying the ethical self to others. This article argues that there is a further element playing out through such practices where alternative food consumption is figured in processes that demarcate social groups. Class is one way through which such boundary formation can come to be understood.

This line of argument has deep roots in the sociology of consumption, from Veblen (1994), Douglas and Isherwood (1996) and Bourdieu (1984) to Slater (1997), Warde (1997) and Bennett et al. (2009), to name but a few. Across the sociology of food there is a great deal of consideration given to the analysis of the social dimensions of eating and differentiation, focused both in and around the home. Studies reported by Murcott (1983), DeVault (1991), Southerton (2002), Bugge and Almås (2006), Mellor et al. (2010), Evans (2011) and Wills et al. (2011) are but a few examples. Moreover, Warde and Martens (2000) consider the symbolic significance of eating out, while Elias (1978) and Mennell (1985) chart such changes in eating over time. Following such lines of inquiry, the article takes a Bourdieusian theorisation of social classification of difference between groups understood as ‘position’, which is taken as an axis around which to understand how and where social actors locate themselves in relation to others, who they are and how they live and want to live within the society in which they operate. Expressions that reveal such positions may be fractured and fragmented, but are nevertheless a means of anchoring an aspect of one’s self in relation to others. Consistent with Bourdieusian methods for recognising positions or indeed class identities we may look to the performance of social practices such as eating, and the acquisition of resources such as food, demanded by these practices. This does not necessarily assume class identification and awareness – class for itself – but considers a class identity as recognisable, among many other possible markers, by preferences for certain cultural goods over others, and the discursive techniques of separation that mark the preferred from the undesirable. The schemes of the habitus operate below the level of consciousness or language (Bourdieu, 1984: 466); for social actors do not create dispositions, worldviews, or indeed ‘habitus’, but acquire them.

What has been less fully explored by the sociology of consumption and the cultural geographies of agri-food networks is the extent to which alternative food consumption in the UK figures in reproducing social, cultural and economic divisions. There is a growing literature in this area (Adams and Raisborough, 2008), but this has mostly focused upon FairTrade consumption in general (Wheeler, 2012). This article draws on insights derived from a case-study involving a two-year period of participant observation at a farmers’ market and at a community food co-operative located within the same neighbourhood of a UK city, developing a sociological perspective with regard to spaces of alternative food consumption. While alternative food supply chains have a long developmental history, with roots in the anti-slavery boycotts of the 1700s and later the co-operative movement (Nützenadel and Trentmann, 2008), it is a contemporary incarnation of the farmers’ market and community food co-op that inspires this study. Here, the valorisation of the embeddedness of local foods (Murdoch et al., 2000), of the connections between producer and consumer (Kneafsey et al., 2008), and the aesthetic of foods positioned as ‘slow’ (Miele and Murdoch, 2002) characterise this the contemporary manifestation of alternative food.

Field notes, 20 in-depth interviews, a survey and documentary analysis reveal how particular ideas about ‘good’, and this case, ‘alternative’ food consumption are used as a means of drawing boundaries between social groups as distinctions are made in talk between foods that are for ‘us’ and those that are for ‘them’. This is noted as particularly characteristic of British ethical consumer movements, which Varul (2009) finds is less pervasive in a German context. Finding such differences across national and cultural contexts may suggest we exercise caution in generalising findings to a wider context. However, Slocum (2008), Guthman (2008a) and Zukin (2008) argue that alternative food initiatives in the US enlist different people in particular moral projects that are socially exclusive and thus ineffectual in bringing about change towards more sustainable ways of procuring, preparing and eating food. While we should expect such cross-cultural variation in the expression of boundary formation and processes of exclusion – such process in the US were grounded in race – this should not deter wider consideration of their implications for the development of equitable alternatives to conventional foodways.

## Distinction: Food for Differentiation

With the onset of post-modernity, consumer choices are thought to be influenced less by social groups and more as the result of self-determination (Harvey, 1990). Questioning the extent to which the consumer experience is characterised by such freedom, Warde (1997) studies the effects of social and cultural change on British food habits between 1968 and 1992, finding persistent and shared patterns of preference and expenditure across groups. This is much akin to Bourdieu’s (1984) seminal study of French eating habits in the 1960s, where necessity imposes a taste for the necessary; working-class meals are ‘elastic’ and ‘abundant’ with avoidance of dishes requiring careful measurement of portions. For the bourgeoisie, the meal marks aesthetic refinement and is premised upon quality over quantity, and style over function. More recently, Lamont (1992) provides a startling portrait of successful French and American upper-middle-class men and their means of forming boundaries between themselves and others, while Southerton (2002) finds that economic, cultural and social resources are central to explaining the accord between respondents in constructing boundaries of separation and alignment between actors. However, Bennett et al. (2009) suggest that while the exercised and cultivated body is an instrument of social classification – through sport, exercise, diet management and maintenance – the practice of eating, particularly within the home, is not found to play a part in this, stating that ‘symbolic distinction finds little place in family dining’ (Bennett et al., 2009: 168). This finding warrants further investigation, particularly given lack of consideration of differences in the form and quality of such foods.

For Sayer (2005b) the privileged have access to goods that are valued by all, and are not only a means of expressing superiority and distinction. A large home does not always simply have an exchange value based solely on conspicuous display, but is also inherently ‘good’, it has a *use* value – it is warm, has enough space to accommodate people and their different needs for sleeping, studying and playing. These are things that matter to people (Sayer, 2011) but, in Bourdieu’s terms, those who are refused such goods come to refuse them. Their exclusion is internalised as a dislike. Alternative food, this article argues, offers one such example of a highly moralised terrain where it is not so simple to argue that a preference for one sort of food is relative to any other. In other words, alternative food may not simply be ‘posh’, it may also have the potential to be ‘good’. Exploring participants’ talk of alterative food and the practices associated with its consumption, the article explores such dynamics of misrecognition, considering how these might come to shape the development of equitable and socially inclusive alternatives to conventional foodways.

### Positioning: Distinction in Quality Foods?

Emerging from the data is an understanding of participants as constructing positions relative to ‘others’. For Bourdieu, ‘it is clear that tastes in food cannot be considered in complete independence of other dimensions of the relationship to the world, to others and to one’s own body, through which the practical philosophy of each class is enacted’ (Bourdieu, 1984: 193). That is, we may gain a sense of our place and that of others within wider relations of order via practical knowledge of resource distribution between groups. Through the habitus, the structures of everyday life interplay, providing unconscious rules that perpetuate its conditions over time. As a structured cultural grammar, the habitus produces a number of possible, although limited, practices. It inculcates ‘reasonable’ and ‘common sense’ social action (Bourdieu, 1990: 55) such as taste, through which we may come to ‘sense or intuit what is likely to befall – and therefore benefit – an individual occupying a given position in social space’ (Bourdieu, 1984: 466). The habitus is the disposition from which one acquires tastes and preferences, from which we can delineate the ‘things and people that go together’ (Bourdieu, 1984: 241). In the way that ‘taste classifies the classifier’ (Bourdieu, 1984: 6) food may bear the signs of vulgarity as well as distinction, but only when perceived relationally. It is through the conscious or unconscious projections that individuals or groups make with regard to these distinctions that class can potentially come to be understood. Building on Bourdieu’s concept of class as a relative position, Southerton (2002), Sayer (2005a), Lawler (2005) and Lamont (1992) consider the moral dimensions of this process. This is significant when considering the practice of ‘alternative’ food consumption, for not only is food in its most conventional form a first need of the human body, but in its ‘alternative’ form, appears to become figured in evaluative judgements about what is good or bad, both morally and aesthetically. By paying attention to what matters to people, we are able to discern normative manifestations of social position, or indeed, class identities, as opposed to commenting solely upon recognition of or affiliation with objective class categories (Payne and Grew, 2005).

The task at hand is thus not classificatory, but an exploration of the dynamics of relational position in the ‘dialectic of competition, distinction and differentiation which is central to symbolic domination’ (Sayer, 2005a: 80). Here, class is considered a relational social position, which can be understood through participants’ normative account of the struggle for resources, namely foods they value in upholding their ways of life. Taste, then, serves the perpetration of symbolic violence as those with the symbolic power to do so judge those who fall short of their ideas of what is right and proper (Skeggs, 2004). Those without symbolic power are ridiculed and shamed for their tastes through these very imaginaries of otherness. Recognising these processes at work is important, for without appreciation of tensions between groups in delineating rules of ‘proper eating’ (DeVault, 1991) we entrench what Hinrichs and Kremer (2002) term a socially homogenised and exclusionary form of sustainable place-making. That is, while Murdoch et al. (2000) suggest that concern for food quality empowers at the local level, this article argues that such a realignment and turn to local embeddedness – as represented by the farmers’ market studied here – can reproduce a balance of power that favours well-to-do consumers (Nygård and Storstad, 1998).

## Methods

Chosen for their situation within the same community, the research sites comprise a farmers’ market (the ‘market’) and a community food co-operative (the ‘co-op’) located on the outskirts of a city in South Wales. The co-op and the market are supported by the same umbrella organisation, yet there is a clear demarcation in their customer profile. A survey (*n* = 300) of customers at both sites provided indicative socio-demographic data as to gender, age, postcode, ethnicity and income, and served mainly to recruit interviewees (see Table 1 for an outline of the dimensions of the sample). While making no claims as to the representativeness of the findings beyond this case, market customers sampled tend to visit from more affluent wards within the county, to earn a net income over £30,000 a year, to have secured postgraduate qualifications, and to have parents who held professional and managerial positions. In contrast, most customers of the co-op live either within the ward or in neighbouring deprived wards (as measured by the Welsh Index of Multiple Deprivation), while 58 per cent earn less that £10,000 per annum, and tend to hold school-leaver qualifications, much like their parents.^1^ Given these marked differences, a case study research strategy suits the imperative to explore the dynamic relationship between these sites, each of which professes to offer the means of connecting consumers with quality food. The market typically comprises 20 stalls staffed by farmers themselves selling produce, meat, and value-added products such as chutneys, pies and cakes. Food and drink is also available for on-site consumption every Sunday morning. The co-op pre-orders fresh produce from a wholesaler and distributes from a local community centre every Tuesday morning. Volunteers then distribute produce into fruit and vegetable bags for collection by local residents who place a further order for the following week.

**Table 1.** List of participants.

Name	CFC/FM	Gender	Age Group	Occupation
**Catherine**	FM	Female	31–40	Manager
**Rhydian**	FM	Male	31–40	Graphic designer
Adam	FM	Male	21–30	Student/works for a charity
**Karen**	FM	Female	51+	University lecturer
Trevor	FM	Male	51+	Retired
**Valerie**	FM	Female	51+	Art student (retired)
Ronald	FM	Male	31–40	Scientist
**Charlie and Sophie**	FM	Male and female Couple	31–40	Both civil servants in local government
Robert	FM	Male	51+	Story-teller
Niamh	FM	Female	21–30	Physiotherapist
**Samantha**	CFC	Female	31–40	Administrator
Julianne	CFC	Female	51+	Unemployed
Bev	CFC	Female	31–40	Administrator
Victoria	CFC	Female	51+	Administrator
Gina	CFC	Female	31–40	Teacher (part time)
**Claire**	CFC	Female	21–30	Massage therapist
**Emily**	CFC	Female	21–30	Unemployed
**Ken**	CFC	Male	41–50	Unemployed
Vera	CFC	Female	51+	Administrator
Sheila	CFC	Female	51+	Sales assistant
Marina	CFC	Female	31–40	Housewife

Interviewees directly quoted in the article are in **bold**.

CFC = Community Food Co-op.

FM = Farmers’ Market.

A sustained period of observation behind the stall-face while ‘participating as volunteer’ (Atkinson and Hammersley, 1994) underscores on-site ethnographic interviews, documentary analysis, survey and 20 in-depth semi-structured interviews lasting between one and two hours, which sought to elicit narratives of everyday food shopping and consumption routines. In speaking their food routines and practices, participants state their position in a field of cultural goods, their likes/dislikes for particular foods and products, and the people that go with them. Due to constraints of space, a proportion of the data gathered from in-depth interviews form the empirical focus of the article, which are chosen because they provide illustrative examples of themes emerging across the data. The article explores 10 participant narratives (five from each site) with the use of pseudonyms to protect the anonymity of interviewees. While narratives elicited from female participants dominate the article, they do so because they capture succinctly the talk of alternative food and its role in reproducing and even destabilising particular ideas of ‘proper’ eating. Reflection upon any potential gendered difference in accounting for taste is best reserved for fuller consideration elsewhere.

## A Field of ‘Alternative’ Food Consumption

### A ‘Hugely Different Attitude to Life’

Exploring means of position-taking, this section introduces five farmers’ market customer narratives. Marking the legitimacy of her food practice, Karen positions herself in contrast to her working-class neighbours. Karen consolidates this distinction by reference to consumer culture more generally, and alternative food politics more specifically. This position is then anchored by articulating her vision of the ‘good life’, thus providing a platform for the pejorative judgement of others. Crucially, explicit categories of class are employed when describing herself in relation to other community members who:

K: … think we’re a bit weird I think.

I: In what way do you think?

K: Just a sort of, you know, ‘middle-class English people’ really, you know [laughter]. This is the sort of working class white [name of City] people who’ve just lived there always, and their families are all linked.

(Karen, market customer)

Rather than claiming ‘ordinariness’ (Savage et al., 2001), Karen judges negatively the stasis of family ties that are historically characteristic of working-class families (Young and Willmott, 1957). This is compounded by later discussions of food practice perceived as characteristic of these very neighbours:

They’re very uncritical about that sort of things I’m criticising in terms of the consumerist culture, cheap food, not worrying about where the stuff comes from how far it’s come or how it’s produced and just saying well something’s cheap and that’s great and bigger cars and going on foreign holidays and thinking that’s you know, taking the attitude that that’s the good life. (Karen, market customer)

Importantly, class differences are not framed as economically determined, for those to whom she refers are believed to have more money than her. They are instead, as Bourdieu argues, separated by degrees of cultural capital; a different idea of the ‘good life’ that involves adopting a critical attitude to cheap food. Defining the ‘good’, Karen contrasts her food politics with working-class consumption practices deemed inadequate, including the preference for cheap food, ‘bigger cars’ and ‘foreign holidays’.

Similarly, Catherine emphasises her commitment to an ethos of *terroir*,^2^ which she claims is practiced by shopping at a farmers’ market, through organic box delivery schemes and eating seasonally. Describing her view of good food is also accomplished by somewhat jovially juxtaposing these foods with those less favourable, and the people that go with them. Indeed, her family eats fresh organic produce, purchased through an organic box delivery scheme or at the farmers’ market, while her friend’s husband ‘was brought up on Findus^3^ pancakes so we always joke about that’. Catherine then goes further by positioning her food preferences in contrast to imagined others, presented as being incapable of delaying gratification:

… but people don’t think anything of going out and spending [money] on pints of cider, or, do you know what I mean, we don’t do, we choose, we don’t do that, we want to go and enjoy our food, and it depends on what people want. (Catherine, market customer)

Here, the imperative to consume in one way rather than another is attributed as a rational choice – ‘it depends on what people want’. Similarly, Valerie’s account of working-class caring practices within the home is cause for some concern. Drawing on her professional experience as a health visitor, Valerie speaks of encounters with working-class families:

The problem *is*, unless it [class] fades away completely you’re going to get an increasing division between those who’ve received lots of health education and have the finances to support it and the sort of under-proletariat who can’t afford it and don’t have that sort of information and don’t believe the information, the problem is *believing* the information […] Breast feeding is a good example erm I can remember erm in [a privileged area] everybody breast feeds for years and years and years. In [a deprived area], everybody thought it was disgusting, you know, absolutely disgusting and I remember one woman said she wouldn’t breast feed because she’d been told if [she] did her breasts would shrivel up, you know. There’s this horror; horror of bodies really. (Valerie, market customer)

While Valerie recognises a ‘huge social class divide in nutrition’, this is attributed to bad choices characterised firstly as information deficit, before moving on to undermine her analysis by invoking visceral responses such as a ‘horror of bodies’ – they *choose* not to believe the information. Moreover, Valerie goes on to speak of families who have ‘allowed their children’s teeth to go black’ in their refusal to accept guidance regarding ‘proper nutrition’, and is similarly astounded that such families do not have ‘cooking pots’. However, despite attempts made to sympathise with structural constraints faced, we detect a discourse prevalent across the interview data with farmers’ market customers: a particular view of the ‘good’ life is upheld in opposition to that which is presented as unsavoury. While Bourdieu argues that expressions of taste betray social position, we see the other side of the coin wherein ‘disgust is one manifestation of a bourgeois project to distinguish the middle class from its others, a means of self-constitution’ (Lawler, 2005: 443).

So far, we see these market customers making some headway in carving out what they consider to make up the rules of ‘proper eating’. Indeed, alternative food as experienced at the farmers’ market is central to framing their ideas about ‘good food’ and the ‘good’ people that partake in its consumption. Problematising this framing, the following section explores a disjuncture between these professed ideas and their practice, highlighting the symbolic violence produced by such misrecognition of class (Skeggs, 2004).

### Disjuncture in Practice: A ‘Good’ Means of Thinking About Food

Despite suggesting that the consumption of quality food from the market forms a core strategy for enacting their ideas of good practice, market customers also speak of the structural constraints faced in doing so. While shopping at the market is presented as ideal, Catherine admits to shopping regularly at a supermarket. Separation from other supermarket customers is accomplished via reflection over what she describes as the trappings of consumer culture, and her ability to see through promotional deals that encourage unplanned purchases:

I think the supermarkets have a very bad effect on local communities in many ways really, but making that critique of it is very hard not to be drawn into it yourself […] and you see all this stuff that you don’t actually need, but because of the way that they market it, you think ‘oh it might be nice to try that’, ‘that looks that’s new that’s different’ or ‘there’s an offer on this’ and you buy all this stuff that you don’t really, you didn’t intend to buy it wasn’t on your list […] ‘oh, well that’s cheap’ you know, ‘shall I get a *golf club*’ or something (laughter) what am I doing! You know. And I think it’s very easy for people to sort of get drawn in to this habit of consumption. Erm, I do get to, you know, buy free range meat at our local butchers occasionally so that’s an option. (Catherine, market customer)

Through such self-critique, these narratives suggest discursive means of critically separating their inevitable engagement with oligopolistic market forces and the ‘others’ who are deemed to engage in such consumer culture due to being simply ‘uncritical of cheap food’. Several participants mark their position by virtue of a claim to a taste for a particular aesthetic and feeling of connection attached to local food (Kneafsey et al., 2008), the enjoyment of the atmosphere at the market and their support for local economic development. Indeed, we see that the embeddedness (Murdoch et al., 2000) of local foods plays into dynamics of distinction, achieved not necessarily by practice, but through a different means of *thinking* about food. That is, the farmers’ market studied here is not so much a space for the purchase of a substantial proportion of household groceries, but a source of enjoyment and occasional indulgence:

Int: So what brings you to this market then to do your shopping?

S: Sunday dinner mostly isn’t it.

C: Yeah, we like the Saturday morning slow meander, do a bit of shopping get a paper, that sort of atmosphere, and yeah, nice Sunday dinner. It’s a weekend treat.

S: Like what we’re doing now.

C: Supplies are running low, we do our shopping on a Monday.

(Sophie and Charlie, market customers)

Taking pleasure in a ‘slow meander’ through the market is common for market customers. What is being problematised is not the enjoyment experienced via a re-engagement with food, especially given the widely perceived disenchantment with a global food supply chain (Nygård and Storstad, 1998), but the unintended consequences for such alternative food developments. That is, the relative success of this market and other similar initiatives such as box schemes and farm shops brings with it the conflation of particular ideas of ‘good’ food with a narrow imagining of alternatives to conventional foodways as less privileged consumers are thought to be beyond the pale of inclusion. That is, the dearth of working-class presence in ‘alternative’ spaces (Guthman, 2008a) is thought to result from financial exclusion (Zepeda, 2009) coupled with disinterest in the wider politics of food.

The following section explores a deeper component of this perceived absence through an examination of talk surrounding food practice among five co-op customers. Just like the market customers, they do not always present a simple class identity, but a narrative of class belonging peppered with references to those from whom they distinguish themselves. Their class position is, then, determined by relational identifications. Much akin to Southerton’s (2002) findings, food continues to figure as a resource, marking what is considered reasonable for ‘us’ relative to ‘them’.

### Subjects of Disgust: Contesting, Resisting and Compensating

Ken identifies as working class by virtue of his belonging to a ‘proper working-class community’, and his work in semi-skilled manufacturing. Being a ‘down to earth working-class guy’ he is separate from the ‘poshies’ who are characterised as the ‘bosses’, and who represents typical market customers:

So when you’re in a room of people and they’re talking a certain way, you know they’re the poshies and they’re the middle classes and you shouldn’t really be there sort of thing you know that’s my feeling. (Ken, co-op customer)

In contrast to Savage et al. (2001), Ken not only articulates an overt class position, but draws a clear boundary between spaces that are ‘for’ one class and not another, reminding us not to throw out the classed baby with the proverbial bathwater. Rather, we can see that there is recourse to support arguments that class identities are expressed through boundary marking (Bourdieu, 1984; Lamont, 1992; Southerton, 2002) between what is considered appropriate for ‘us’ and ‘them’:

I went round there [the market] one time, like, because I thought they’d have interesting food and stuff, but it was really quite expensive a lot of the stuff it was quite strange I mean even though it’s in the [local] area. Most of the people there were quite middle class and it seemed more aimed towards them than the local people […] I bought one or two things there but they were just, there was a lot of people there wearing afghan stuff and ‘hello Rodney’ and all this you know [laughs] and then there’s me with my [local] accent like, there wasn’t much evidence of a [local] accent there like you know and you did feel like, what’s this doing? This is not for me, you know. (Ken, co-op customer)

Crucially, the middle-class ‘poshies’ are identified as the typical customer, inciting his discomfort with this space. Ken also resists the moralising discourse that Skeggs (2004) theorises as a reproachful middle-class gaze. In mocking the accent of market customers, Ken implies that the overt display of a sociality unfamiliar to him effectively undermines what he thought this market to be – ‘what’s this doing?’ The market is not a space he recognises, it is not part of his cultural repertoire and therefore it is not considered ‘for’ him. In a similar way, Emily dismisses the benefits of consuming organic food by referring to scientific controversies. This dismissal is, however, blemished by admission that she would be ‘more conscious’ if she were financially able. The structural inhibitions Emily faces are thus compensated through a narrative of scepticism. Under this thick skin of compensation, Emily conflates ‘exchange’ with ‘use’ value (Sayer, 2005a), dismissing what may be ‘good’ and refusing what she is refused:

A younger friend, they are quite well off financially, erm, so when I go there now she’ll like ‘oh you know what Helena says about this, well of course this has to be organic’, yeah well if I’d had that, then maybe I would be more conscious about it, but I can’t be, so let’s not worry too much. At the end of the day we could all get sick through hormones in meat or whatever, but if it happens it happens. And I, I don’t think I’m condemning myself by eating your normal, your average chicken or things like that. (Emily, co-op customer)

It is not so simple to say from this that Emily is a disengaged ‘other’ who is not interested in food, but that she has limited capacity to engage with alternative food as currently defined. Claire, also a co-op customer, explains her position on healthy eating, patently pointing out that speaking of such efforts is not an attempt at displaying that she is any ‘better than anyone else’, but suggests she is ‘fussy’ and ‘suspicious’ of pre-packaged meals. Indeed, Claire emphasises that she does not judge those who do not cook from scratch, for she used to eat microwave meals herself: ‘you have to, life is manic’. Now, she nips in and out of local shops to secure bargains, and purchases meat from a local restaurant supplier. Through connections with friends and family, she provides at least one hot meal a day for herself and her daughter at an affordable price. The co-op presents opportunities for Claire to obtain affordable vegetables, and is also an occasion for her four-year-old daughter to be introduced to a world of food outside of supermarkets. At the co-op, she is able to resolve a perceived contradiction between differing qualities of food:

All you can really do is try and shop as locally as possible to stop us ending up with a situation where everything is out of town. We do, I mean we should probably care a bit more about organic stuff than we do but I’m a bit cynical in my old age! I don’t trust many retailers and I think unless you’re growing it yourself then you can’t really know that it is organic, sometimes I think in the supermarkets, I think why am I buying these organic runner beans form Kenya when I don’t know how many air miles and stuff … there are just so many things you’re supposed to care about aren’t there! (Claire, co-op customer)

Contrasting the imagery of working-class consumers put forward by market customers, supporting local enterprise is a theme that occurs across market and co-op customer narratives. Frequenting many different stores, knowing when fresh food will be reduced in price, and where they may secure the best value for their money does not resonate with the imagery of a disengaged food consumer. However, while as a public space the market does not refuse access to anyone per se, the interplay of social, cultural and economic dynamics combine, excluding some by fostering a feeling of discomfort. As Ken suggests, the absence of local accents and the sense that many of the other customers are acquainted brings with it a feeling of not belonging. Being excluded on the grounds of price is, then, but one part of this story.

Furthermore, Samantha grows her own food, a practice rooted in family tradition passed on from her grandfather. When speaking of composting waste and growing fruit and vegetables, she not only provides rebuttal to the imagery of the ‘disgusting’ (Lawler, 2005) working-class subject, but positions *her* way of engaging with contemporary ‘food politics’ (Micheletti and Stolle, 2007) as more genuine than her middle-class counterparts, whom she suggests engage merely ‘to be trendy’. In laying the foundations for this appeal, Samantha brands ‘alternative’ consumers as ‘worthies’; people who display and preach of their political efforts. Crucially, these ‘worthies’ are defined as those whom she imagines see themselves as superior to her position as a ‘working-class girl’:

I think there’s a whole kind of group of hidden green people who don’t wear hemp and kind of […] they’re not overtly green, who make green choices *most* of the time but are quite pragmatic about it. I think I probably fall in that camp but you know […] we don’t kind of wear it on our sleeve literally. And you know therefore I just think I’m not going as far as some other people who are out there in kind of kaftans and long skirts and beads […] there’s a guy I used to work with that really is quite green and […] he was quite critical and I was like ‘for goodness sakes … I compost, and you’re criticising me for using a water-based paint with a tiny bit of solvent it in you know … get a grip’ so yeah he was interesting, one of those annoying types who’s just *way* too worthy! (Samantha, co-op customer)

Recounting further experience of ‘worthy’ others, Samantha speaks of the ‘yoga Mums’ who contravene her pragmatic and private engagement with ‘green’ issues. While not speaking directly to issues of food, Samantha employs these examples in illustrating her position as a pragmatic rather than a ‘worthy’ consumer. Indeed, it is through this narrative – outlining her experience of this ‘worthy’ gaze – that she accomplishes her account of growing food and composting as a rebuttal to judgements made by those whom she suggests are concerned with display over substantive action:

She [a ‘Yoga Mum’] gets a bit kind of full on about re-usable nappies which is a bit of a cheek because she’s only just started to use them […] and well some of us have been using them quietly without making a big deal about it for months! […] When she makes a green decision everybody knows about it and all of a sudden any other way is not right. (Samantha, co-op customer)

This appeal to authenticity (Vannini, 2009) acts as a tool for rebuttal, resisting the imagery of the inadequate consumer by pointing to the artificiality of the market. Skeggs (2004) might argue this is a tool of anti-pretentiousness, of refusing what we are refused. Rhydian is similarly critical of the farmers’ market as a space for conspicuous performance of ideals, a claim he makes from observations as an occasional customer himself:

It just feels stereotypical, people make an effort to go on their bikes, and I don’t know many of those people go everywhere on their bikes or whether they just feel like ‘*oh you should*’ and you take your plastic bags there and your recycled bags and make a bit of an effort there, and I don’t know maybe if people carry that on in everyday of their life […] It just feels a bit artificial I suppose, but maybe I’m just looking at myself, and everyone else lives their lives like that all the time. (Rhydian, market and co-op customer)

While such competing claims are part of the scene of everyday life and talk, with actors having what Potter (2010) terms their ‘stake’ in the matter of ‘good food’, this article seeks not to adjudicate such claims. The analysis serves to highlight spaces of alternative food consumption as such a contested and classed space, pointing to consequences for future alternative food developments. Indeed, is it so simple that excluded consumers are content to remain so, on the basis that they are refusing an affectation? This is problematised by the notion that somewhere underneath the ‘thick skin’ of compensation (Southerton, 2002) ‘alternative’ food is valued. The ways in which it has been appropriated by middle-class consumers, however, is not. In Sayer’s (2005b) words, they may not be merely refusing what is ‘posh’ but also what is ‘good’.

## Conclusions: Separating the ‘Posh’ from the ‘Good’

To recap, market customers present imagery of others as not wishing to engage with ‘alternative’ food practice as they are instead perceived as committed to a conspicuously consumptive vision of the ‘good life’ – they make *choices* between spending money on ‘pints of cider’ or ‘good food’. Following the analysis of data presented above, we might consider another explanation for their absence: their discomfort with what is perceived as a space not ‘for them’ (Southerton, 2002). For market customers such as Sophie and Charlie, the market provides a space for sociality, while it is considered both laughable and uncomfortable for co-op customers such as Ken and Samantha. Although middle and working-class consumers each find some fault with the practices of the ‘other’ as either repugnant or ridiculous, we should take seriously that it is the derision expressed by middle-class consumers that goes hand-in-hand with the association of ‘good’ food with *their* food practices. It is this very insistence that resounds in the development of ‘alternative’ food initiatives such as the market studied here, driving a narrow view of future alternatives, which Guthman and DuPuis (2006) suggest reproduces a white, middle-class ‘politics of perfection’. Having explored the accounts of co-op customers, it may not even be so simple as to suggest that working-class consumers may feel excluded culturally, but the compensatory strategies (Southerton, 2002) developed to cope with such exclusion render the idea of developing one all-encompassing strategy for ‘alternative’ food provision as incongruous; an argument I develop elsewhere more fully (Paddock, 2015).

Indeed, while Bourdieu (1984) argued that it is common for those who are refused certain goods to refuse what they are refused, it is not unreasonable to imagine the future refusal of alternative food developments. In refusing these goods that are culturally legitimised, they ‘not only invite the scorn of the dominant in so doing, they also confirm their refusal of internal goods which are valuable regardless of whether the dominant happen to value them’ (Sayer, 2005b: 121). Of sociological note here is that to imagine an equitable and sustainable food future, we might suggest that the ‘posh’ be separated from the ‘good’ by taking seriously the difference between ‘use’ and ‘exchange’ value in alternative food initiatives. Some goods are valuable not simply because others regard them so, but are ‘good’ because they offer nutrition, shelter or comfort. Recognising the field of alternative food consumption as a highly differentiated terrain may encourage a shift towards imagining alternatives that do not appeal solely to a middle-class realm of cultural comfort. To pursue strategies that create separations between what is considered food for ‘us’ and food for ‘them’ (Southerton, 2002) is to continue to misrecognise class in a way that secures, for the more privileged, practical advantages of the field of ‘alternative’ food consumption, of what is ‘good’ and not what is simply ‘posh’. This is not to suggest that markets and community co-operatives do not have significant roles to play in providing a platform for community sociability and for providing a means to access fresh food. Rather, it remains to recognise whose communities are being served, whose are excluded, and to what effect.

Taking on board a critical amendment of Bourdieu’s approach as proposed by Sayer (2005a), this article considers class as a normative expression of position. That is, in exploring what *matters* to people, we are able to discern normative manifestations of class culture, rather than commenting only upon affiliation with class categories (Payne and Grew, 2005). Not only a first need of the human body, the moralised terrain surrounding consumption lays bare the highly charged nature of food as a carrier of our culture (Douglas and Isherwood, 1996), embodying more or less what Sennett and Cobb (1972) describe as the hidden injuries of class. In moralising the ‘other’ for not consuming ‘good’ food, we misrecognise the harsh realities faced by many in feeding themselves and their families. Indeed, watching and listening to people go about their food shopping, it becomes clear that food carries along with it the hopes and desires about how we would like to live, while the imagined food practices of ‘others’ can become the subject of pride, envy, discomfort and derision. Competing claims as to the authenticity of one’s food practice make fertile ground for further research.

In this way, a Bourdieusian conceptualisation of ‘position’ is useful in highlighting the nuances of uptake and discomfort at work in a field of alternative food consumption. Exploring such nuances, albeit through one case study, begins the process of recognising class difference and its role in mediating the development of alternative, more sustainable and inclusive food practices for the future. This article provides some armoury to this call, and points towards a future research agenda concerned with exploring the potential to realise a more reflexive form of localism (DuPuis and Goodman, 2005) that takes into account the diversity of positions, tastes and worldviews that shape what may come to be considered reasonable food. Indeed, we may move beyond a limited ‘politics of the possible’ (Guthman, 2008b), recognising that farmers’ markets provide a means of engaging predominantly white middle-class consumers. It might not be sufficient to expect that diverse populations can or will engage on these same terms. Recognising this dynamic is one step in lifting the symbolic violence governing alternative food developments. As Lawler (2005: 443) observes, ‘It is important, in other words, to challenge an unmarked and unproblematized middles classness which claims a monopoly on true humanity’.

## Notes

1. Space for discussing the survey data is limited, and given the intention of this article to explore position-taking through talk, survey data briefly introduces the socio-demographic composition of each site.

2. ‘Terroir’ indicates the specificity of place, and often translates into the practice of matching the foods and wines of a region’s complete environment.

3. A brand of frozen convenience food.
